# Longitudinal Trends and Predictors of Medicaid Utilization Among Middle-Aged and Older Adults

**DOI:** 10.1093/geroni/igaa057.259

**Published:** 2020-12-16

**Authors:** Jane Tavares, Marc Cohen

**Affiliations:** 1 University of Massachusetts Boston, Florence, Massachusetts, United States; 2 University of Massachusetts Boston, Boston, Massachusetts, United States

## Abstract

Over fifteen million older adults in the United States rely on the means-tested Medicaid program for healthcare coverage (accounting for approximately 20% of total Medicaid beneficiaries according to the Centers for Medicare and Medicaid Services). With the older adult population growing exponentially, there has been concern that steadily rising Medicaid spending will skyrocket among this group and that there may be a need to reconfigure coverage of the program. However, few studies have longitudinally examined which factors are related to utilization of the program over time among older adults in order to better understand how any future coverage changes might impact this group. This study used the 1998 to 2014 waves of the Health and Retirement Study (N=8,162) to analyze a representative sample of those 50 and older regarding demographic, health, and economic trends associated with Medicaid utilization over a sixteen-year period. Descriptive analyses showed stable longitudinal patterns such that those who utilized Medicaid had significantly poorer health and fewer financial resources compared to those who never accessed Medicaid. Multivariate analyses further revealed those who were older, female, minority race/ethnicity, less educated, in poorer health, below the federal poverty line, and with lower net wealth had a higher proportional risk of utilizing Medicaid during the observed time period than their counterparts. The findings from this study highlight the importance of monitoring changes in the documented risk factors over time in terms of their impact on Medicaid utilization and underscore the need to consider how these factors may be interrelated.

